# Extra Eight Pages

**Published:** 1868-08-01

**Authors:** 


### *Extra Eight Pages.*

Eight pages are added to the present number. The press of valuable original and translated matter is such that we must still beg pardon of correspondents whose articles are delayed in appearance. A large number of papers on *Shoulder Presentations*, we have placed in the hands of a competent friend, who promises us a digest of them, with general practical comments. The article promised on the new process of preserving the *cadaver*, was crowded out of this number, and as it has been generally published in other medical journals, will not be published in this, unless its insertion is especially requested.

